# Rethinking the Intrinsic Sensitivity of Fungi to Glyphosate

**DOI:** 10.3390/biotech11030028

**Published:** 2022-07-26

**Authors:** Tuomas Tall, Pere Puigbò

**Affiliations:** 1 Department of Biology, University of Turku, 20500 Turku, Finland; teltal@utu.fi; 2 Nutrition and Health Unit, Eurecat Technology Center of Catalonia, 43204 Reus, Catalonia, Spain; 3 Department of Biochemistry and Biotechnology, Rovira i Virgili University, 43003 Tarragona, Catalonia, Spain

**Keywords:** glyphosate, herbicide, domain architecture, fungi, multi-domain enzymes, enzyme, sensitivity, shikimate pathway

## Abstract

The 5-enolpyruvylshikimate 3-phosphate synthase (EPSPS) is the central enzyme of the shikimate pathway to synthesize the three aromatic amino acids in fungi, plants, and prokaryotes. This enzyme is the target of the herbicide glyphosate. In most plants and prokaryotes, the EPSPS protein is constituted by a single domain family, the EPSP synthase (PF00275) domain, whereas in fungi, the protein is formed by a multi-domain structure from combinations of 22 EPSPS-associated domains. The most common multi-domain EPSPS structure in fungi involves five EPSPS-associated domains of the shikimate pathway. In this article, we analyze 390 EPSPS proteins of fungi to determine the extent of the EPSPS-associated domains. Based on the current classification of the EPSPS protein, most fungal species are intrinsically sensitive to glyphosate. However, complex domain architectures may have multiple responses to the herbicide. Further empirical studies are needed to determine the effect of glyphosate on fungi, taking into account the diversity of multi-domain architectures of the EPSPS. This research opens the door to novel biotechnological applications for microbial degradation of glyphosate.

## 1. Introduction

Glyphosate-based products are the herbicide most used against weeds worldwide. The herbicide targets an almost universal enzyme in plants, the 5-enolpyruvylshikimate-3-phosphate synthase (EPSPS), also known as aroA [1,2]. The EPSPS is a key enzyme in the shikimate pathway for the synthesis of Tyrosine, phenylalanine, and tryptophan [3]. Because the *epsps* gene is not found in animals, the use of glyphosate is supposed to be safe for human health. However, the *epsps* gene is also present in most fungal and bacterial species. Thus, glyphosate may have an effect on microbial communities of free-living and host-associated microorganisms [4,5,6,7]. Recent studies of the EPSPS-glyphosate relationship have provided clues on the potential effect of the herbicide on the microbiota [8]. A comprehensive analysis of the potential sensitivity of the EPSPS protein has shown the potential impact of the herbicide on several species of plant, fungi, and bacteria [4,5] (e.g., the herbicide has the potential to affect half of the human gut microbiota [4]). The current classification of the EPSPS enzyme includes four EPSPS classes (class I: potentially sensitive and class II–IV: potentially resistant) based on amino acid markers in the EPSPS single-domain protein characteristic of plants and bacteria. However, the multi-domain EPSPS structure in fungi may lead to a complex response to the herbicide that has been largely overlooked.

Here, we analyze the evolution of the EPSPS domain in fungi and the distribution of additional EPSPS-associated domains. The EPSPS enzyme, at least in its single-domain structure characteristic of plants and prokaryotes, closes after its interaction with the two substrates, shikimate 3-phosphate (S3P) and phosphoenol pyruvate (PEP) [9]. Most of the EPSPS protein structures in the Protein Data Bank are in the closed form [10], and there are no representatives of any multi-domain EPSPS structure characteristic of fungi. The target-site sensitivity to glyphosate, also known as intrinsic sensitivity, was estimated based on the presence of amino acid markers in the EPSPS active site [4]. In addition, there are non-target-site factors (e.g., levels of gene expression of the *epsps* gene) that highly contribute to modulating the response of organisms to the herbicide [11,12,13]. The intrinsic sensitivity of the EPSPS to the herbicide has been largely studied in bacteria [4,5,14], and the results are in agreement with empirical microbiome studies [15,16,17,18,19]. Although more than 90% of fungal species have been classified as potentially sensitive to glyphosate (*n* = 789; 726 sensitive, 6 resistant, and 57 unclassified) [4], the response of a fungal multi-domain EPSPS to the herbicide glyphosate is yet unclear. The results of our survey of EPSPS-associated domains in fungi will help determine the effect of glyphosate on fungal species. Moreover, finding intrinsically resistant fungal strains is relevant in the development of agro-biotechnological applications to identify novel strategies for microbial degradation of glyphosate.

## 2. Materials and Methods

### 2.1. Dataset

EPSPS-associated domains present in EPSPS proteins were obtained from the PFAM (http://pfam.xfam.org, accessed on 8 July 2022), a comprehensive database of protein domains [20]. The dataset of EPSPS proteins was gathered from https://ppuigbo.me/programs/EPSPSClass, accessed on 20 July 2022 [4], and included 1175 EPSPS proteins with multi-domain structure prokaryotes and eukaryotes. The dataset included a subset of 390 out of 422 fungal proteins with multi-domain EPSPS structure (Supplementary Table S1).

### 2.2. Bipartite Network

A bipartite network of protein domains in fungal species was built with the program Cytoscape [21]. This network was used to visualize the presence and absence of EPSPS-associated domains and the distribution of the different architectures of the multidomain EPSPS protein in fungi.

### 2.3. Phylogenetics Analysis

EPSPS domains, from the dataset of EPSPS protein sequences of fungi, were aligned with the programs MUSCLE [22] and curated with Gblocks [23]. The program FastTree2 [24] was used to build a phylogenetic tree of the EPSPS domain. We utilized Dollon parsimony with the program Count [25] to analyze the evolution of the EPSPS-associated domains in fungi.

### 2.4. Potential Sensitivity to Glyphosate

The potential sensitivity to glyphosate was estimated using the EPSPSClass web server (https://ppuigbo.me/programs/EPSPSClass, accessed on 20 July 2022) [4]. EPSPS proteins are currently divided into four main classes (class I, sensitive; class II–IV, resistant).

## 3. Results and Discussion

### 3.1. Functional Characterization of EPSPS-Associated Domains

EPSPS proteins were defined by the EPSPS domain, which is approximately 1350 nucleotides long (450 amino acids) [4]. However, there were variations in length of the EPSPS protein, depending on the total number of EPSPS-associated domains and ranges between 163 (A0A101J2R9_9PORP) and 3206 (A0A094CHT8_9PEZI) amino acids. A multi-domain EPSPS structure was observed in most fungi, but it was rarely observed in plants and bacteria. Usually, multi-domain EPSPS genes of bacteria and plants are formed by two domains, whereas the fungal EPSPS is a larger sequence composed of more than five EPSPS-associated domains. Although most of the EPSPS-associated domains are involved in the shikimate pathways for the synthesis of the aromatic amino acids, promiscuous domains were also present (Table 1 and Supplementary Table S2).

The EPSPS-associated domains can be classified into four main functional categories: shikimate (involved in shikimate pathway proteins), enzymes (proteins with catalytic function), expression (domains involved in gene expression), and structural function (proteins that do not have a catalytic function, such as binding sites, histones, and helix-turn-helix domains). The distribution of the EPSPS-associated domains in a dataset of 1175 multi-domain proteins showed certain dominance of the domains EPSP_synthase (as a marker of the EPSPS proteins), Shikimate kinase (SKI), 3-dehydroquinate synthase (DHQ_synthase), 3-dehydroquinate dehydratase (DHquinase_I), and Shikimate dehydrogenase substrate binding domain (Shikimate_dh_N; Table 1 and Supplementary Table S2). In some proteins, the multi-domain structure of the EPSPS included more than one hit to the EPSPS domain in pfam (e.g., S8DP49_FOMPI and A0A067M4R2_9AGAM).

### 3.2. Distribution of the EPSPS-Associated Domains in Fungi

**Figure 1:**
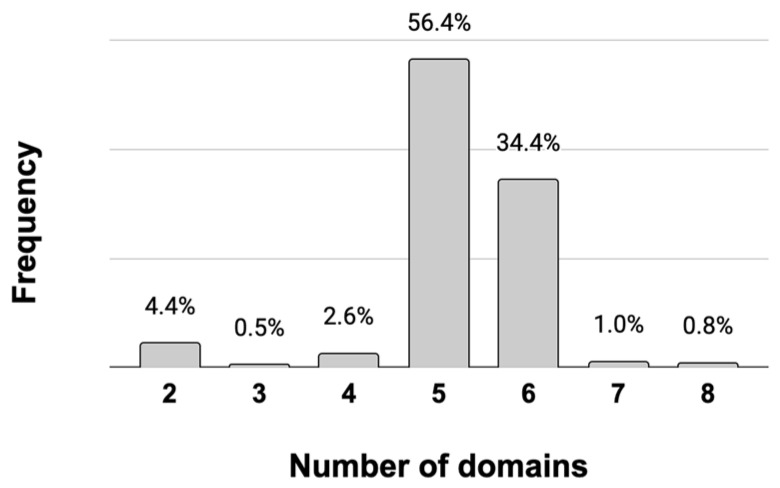
Frequency of multi-domain structures of the EPSPS in fungi. There are 390 out of 420 EPSPS proteins with multi-domain structure in fungi. The majority of these proteins have five or six domains.

Most of the EPSPS-associated in fungi were involved in the shikimate pathway (e.g., SKI, DHQ_synthase, DHquinase_I) and in the synthesis of aromatic amino acids (e.g., Shikimate_dh_N, PDH). There were also some promiscuous domains (e.g., HTH_3) associated with the EPSPS in some fungal species. Infrequent, but amply distributed, EPSPS-associated domains in fungi were involved in DNA modification and gene expression. A total of 22 domains were present in diverse domain architectures of the EPSPS protein, across 390 fungal species (Supplementary Table S3). However, in fungi, the most common multi-domain structure of the EPSPS consisted of five EPSPS-associated domains (Figure 1), mostly involved in the shikimate pathway, such as SKI (*n* = 374); DHQ_synthase (*n* = 374); DHquinase_I (*n* = 367); Shikimate_dh_N (*n* = 366); and Shikimate/quinate 5-dehydrogenase (Shikimate_DH; *n* = 136). In fungi, 16 out of 22 EPSPS-associated domains were only present in less than three proteins (Supplementary Table S3).

**Figure 2:**
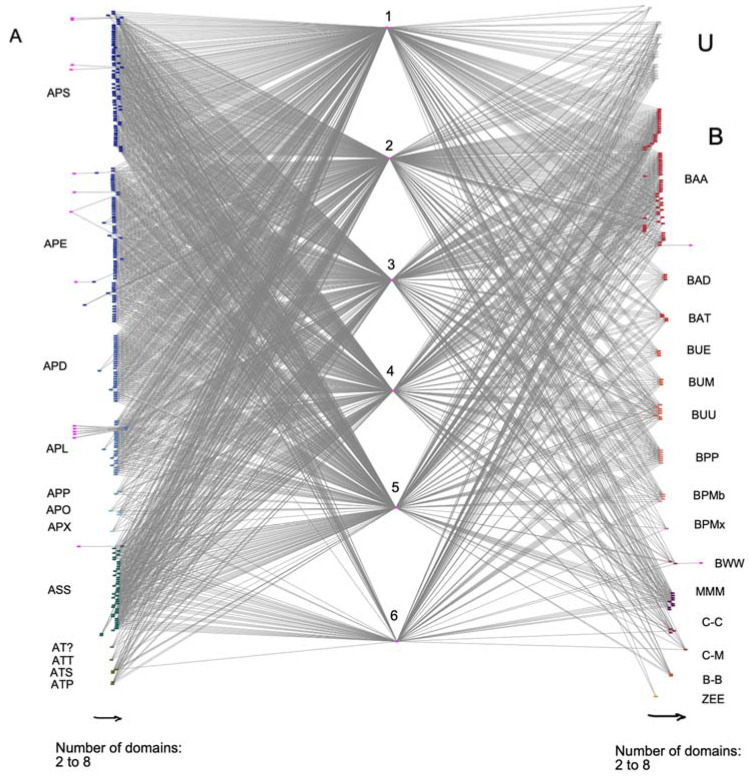
A bipartite network of EPSPS-associated domains in fungal species. Letters stand for different phylums and classes of fungi. On the left side, there are fungi of the phylum ascomycota (A), and on the right side, there are fungi of phylum Basidiomycota (B), Mucoromycota (M), Chytridiomycota (C), Blastocladiomycota (Bl), Zoopagomygota (Z), and Unknown taxa (U). Numbers correspond to fungal multi-domain: (1) DH synthase, (2) EPSP synthase, (3) SKI, (4) DHquinase_1, (5) Shikimate_dh_N, and (6) Shikimate_DH. Arrows on the bottom of the figure note the number of domains in the multi-domain, which ranges from two to eight domains. Subphylum and class starting from the upper left are Pezizomycotina: Sordariomycetes (APS), Eurotiomycetes (APE), Dothideomycetes (APD), Leotiomycetes (APL), Pezizomycetes (APP), Orbiliomycetes (APO), and Xylonomycetes (APX); Saccharomycotina: Saccharomycetes (ASS); Taphrinomycotina: incertae sedis (AT?), Taphrinomycetes (ATT), Schizosaccharomycetes (ATS), and Pneumocystidomycetes (ATP); Agariomycotiina: Agaricomycetes (BAA), Dacrymycetes (BAD), and Tremellomycetes (BAT); Ustilaginomycotina: Exobasidiomycetes (BUE), Malasseziomycetes (BUM), and Ustilaginomycetes (BUU); Pucciniomycotina: Pucciniomycetes (BPP), Microbotryomycetes (BPMb), and Mixiomycetes (BPMx); Wallemiomycotina: Wallemiomycetes (BWW); Mucoromycotina: Mucoromycetes (MMM); Chytridiomycota: Chytridiomycetes (C-C) and Monoblepharidomycetes (C-M); Blastocladiomycota: Blastocladiomycetes (BI-B) and Entomophthoromycotina: Entomophthoromycetes (ZEE).

We have analyzed the distribution of the EPSPS-associated domains in different taxonomic groups of fungi (ascomycota, basidiomycota, mucoromycota, chytridiomycota, blastocladiomycota, zoopagomycota) (Figure 2). Ascomycota was the most variable phylum in terms of EPSPS-associated domains and contained several infrequent domains. Moreover, the least number of Shikimate_DH domains were present in ascomycota (e.g., this domain was least prevalent in Eurotiomycetes, Dothideomycetes, and Leotiomycetes). Most multi-domain architectures (*n* = 220) contained structures with five domains involved in the shikimate pathway, and approximately 1/3 of the protein sequences (*n* = 134) had all six domains of the shikimate pathway. Thus, the overall trend in fungal EPSPS proteins, as shown in the bipartite network, was an association of the EPSPS domain with other domains of the shikimate pathway.

### 3.3. Phylogenetics Analysis of the EPSPS Protein in Fungi

**Figure 3:**
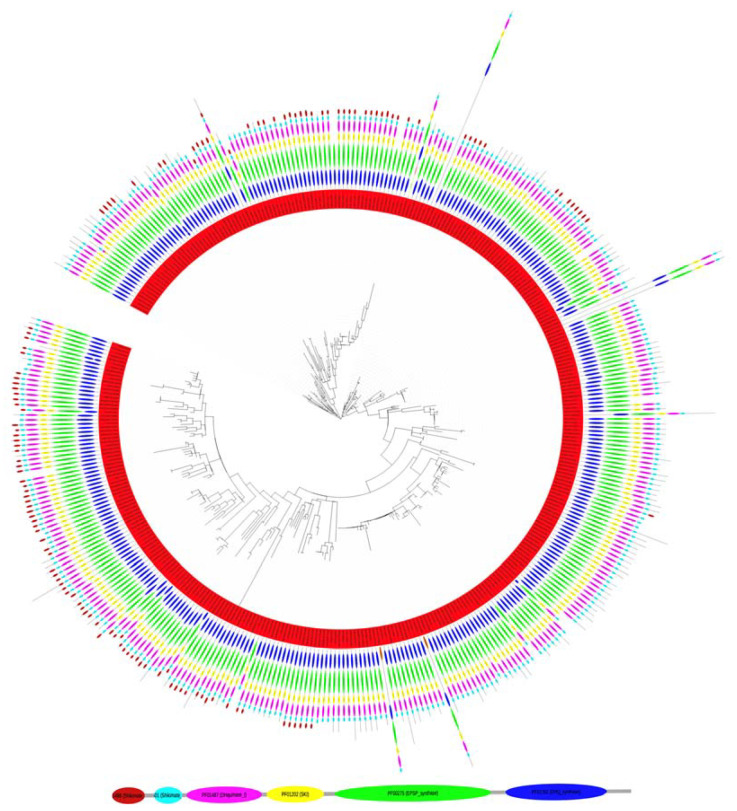
Phylogenetic conservation of the multi-domain structure of the EPSPS protein in 390 fungi. The ancestral domain structure includes the EPSPS (PF00275; green) and EPSPS-associated domains Shikimate_DH (PF01488l; maroon), Shikimate_dH_N (PF08501; light blue), DHQuinase_I (PF01487; pink), SKI (PF01202, yellow), and 3-dehydroquinate synthase (PF01761; blue). Figure modified from [4] with permission. A detailed view of the phylogenetic tree is freely accessible at https://itol.embl.de/tree/13023210641470501567596434, accessed on 20 December 2021.

The EPSPS multi-domain structure in fungi was heterogeneous across the phylogenetic tree. However, most of the species had five or six domains in the EPSPS protein (Figure 3). Our analysis indicated that a protein sequence with all six most abundant domains (i.e., a six-domain multi-domain structure) was the original EPSPS sequence in fungi (Table 1). Thus, the majority of sequences with five multi-domain structures raised by loss of the Shikimate_DH independently in different branches of the evolutionary tree. Moreover, many domains have been independently lost at early and late stages in the evolution of fungi. Notice that in some sequences, the Shikimate_DH (located at the C-term of the EPSPS protein) was disrupted. We speculate that in some cases, this domain was lost in a crossover event without affecting the functionality of the shikimate pathway. Moreover, we do not know if the domain function was preserved in a different protein. On the other hand, the Dollon parsimony analysis of the fungal phylogeny (Figure 3) indicated that infrequent domains were late inclusions into the multi-domain structure.

### 3.4. Potential Sensitivity to Glyphosate in Fungi

Here, we analyzed the frequency and evolution of EPSPS-associated domains to determine variations in the intrinsic sensitivity of the EPSPS protein to glyphosate. In bacteria and plants, the EPSPS protein sequence has a single domain, whereas fungal EPSPS proteins contain several domains [4]. Therefore, the EPSPS protein folding in fungi may result in a different interaction with the herbicide compared to the plant and bacteria EPSPS [8]. These potential effects of the multi-domain structure of the EPSPS have been mostly neglected. Moreover, additional non-target mechanisms of resistance (e.g., efflux pumps, vacuolar sequestration, and metabolization of glyphosate) or sensitivity (e.g., toxic effect on the mitochondria) to glyphosate modulate the intrinsic sensitivity status in the EPSPS protein [8,27] and may have a differential effect on fungal species. Several experimental and field studies have shown a negative effect of glyphosate on fungal communities in soil [28] and underground host-associated interactions [29]. Other fungi have developed non-target site resistance mechanisms (e.g., *Purpureocillium lilacinum* is able to degrade glyphosate and use glyphosate as a nutritional source [30]). The EPSPS of *P. lilacinum* (PWI66746.1) is sensitive to glyphosate. Moreover, it has been suggested that the carbon-phosphorus bond in glyphosate is the major metabolic degradation mechanism utilized by fungi [31].

**Figure 4:**
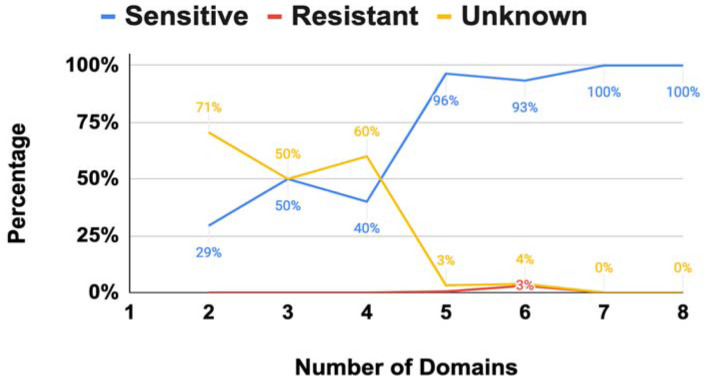
Distribution of sensitive, resistant, and unknown EPSPS sequences to glyphosate.

The EPSPS is a two-substrate enzyme with an open (without ligand) and closed (with ligand) conformation. Glyphosate’s mode of action is competitive against the PEP and noncompetitive against the S3P [9]. However, the dual conformation of the EPSPS has been mostly studied in single-domain proteins of plants and bacteria; thus, its effect in a multi-domain structure is quite uncertain [8]. Our results showed that 354 (90.8%) fungi were potentially sensitive to glyphosate, 5 (1.3%) were resistant, and 31 (7.9%) were unknown (i.e., unclassified EPSPS proteins based on the current classification system). Interestingly, all EPSPS resistant species were class III, a not yet fully understood mechanism of resistance to glyphosate only present in a very small fraction of species [4]. However, the general trend changed depending on the number of domains (Figure 4). Multi-domain structures of the EPSPS protein with less than five domains had a significantly larger amount of unclassified sequences (Figure 4). Thus, further experimental evidence and new models are needed to determine the sensitivity of fungal organisms to glyphosate.

## 4. Conclusions

In fungi, the most common multi-domain structure of the EPSPS ranges from two to eight domains. The ancestral state of the EPSPS protein included six domains (DHquinase_I DHQ_synthase, EPSPS, SKI, Shikimate_DH, and Shikimate_dH_N), as shown in the phylogenetic analysis. The wide diversity of EPSPS multi-domain structure in fungi is the product of several independent rearrangements of domains throughout evolution. Analyses of the EPSPS enzyme showed that most fungi are potentially sensitive to glyphosate. However, the total number of EPSPS-associated domains have an effect on the potential sensitivity status. Future analyses will be necessary to determine how different EPSPS multi-domain architectures affect the sensitivity of the EPSPS enzyme to glyphosate. These studies may have a substantial contribution to the development of novel biotechnological applications for microbial degradation of glyphosate.

## Figures and Tables

**Table 1:** Most common functions of the EPSPS-associated domains.

Domains	Freq	Sp	D	Function
EPSPS	1448	8249	111	Shikimate pathway (SP), EPSP Synthase
SKI	424	8075	171	SP, phosphorylates shikimate
DHQ_synthase	420	7663	95	SP, removes a phosphate from DHAP
DHquinase_I	416	2247	135	SP, 3-dehydroquinate dehydratase
Shikimate_DH_N	402	7829	185	The substrate binding domain of the shikimate dehydrogenase
HTH_3	218	9596	752	A major structural motif capable of binding DNA (Helix-turn-helix)
Shikimate_DH	160	6879	139	SP, quinate 5-dehydrogenase
PDH	127	1584	1551	Part of tyrosine biosynthesis (Prephenate dehydrogenases)
Cytidylate_kin	88	6928	37	Kinase of cytidine 5’-monophosphate
PF13193	17	8190	3379	AMP-binding enzyme C-terminal domain for PF00501

Domains: domain names; Freq: frequency of the domain in the subset of proteins with more than one domain; Sp: number of species in the pfam database (on May 2022) with at least one copy of the domain; D: number of domain architectures in pfam database (on May 2022); and Function: description of the product. This table has been modified with permission by authors from [26].

## Data Availability

A dataset of pre-computed EPSPS proteins is freely available from the EPSPSClass web server at http://ppuigbo.me/programs/EPSPSClass/, accessed on 20 July 2022.

## Supplementary Materials

The following supporting information can be downloaded at: https://www.mdpi.com/article/10.3390/biotech11030028/s1. Table S1: List of EPSPS proteins with more than one domain in fungi analyzed; Table S2: List of EPSPS-associated domains; Table S3: List of EPSPS-associated domains in fungi.
